# DPI_CDF: druggable protein identifier using cascade deep forest

**DOI:** 10.1186/s12859-024-05744-3

**Published:** 2024-04-05

**Authors:** Muhammad Arif, Ge Fang, Ali Ghulam, Saleh Musleh, Tanvir Alam

**Affiliations:** 1 College of Science and Engineering, Hamad Bin Khalifa University, Doha, Qatar; 2 State Key Laboratory for Organic Electronics and Information Displays, Institute of Advanced Materials (IAM), Nanjing 210023, P. R. China, Nanjing 210023, China; 3 Information Technology Centre, Sindh Agriculture University, Sindh, Pakistan; 4 Center for Research Innovation and Biomedical Informatics, Faculty of Medical Technology, Mahidol University, Bankok, 10700 Thailand

**Keywords:** Druggable proteins, Bioinformatics, PSSM, Physicochemical features, Cascade deep forest

## Abstract

**Background:**

Drug targets in living beings perform pivotal roles in the discovery of potential drugs. Conventional wet-lab characterization of drug targets is although accurate but generally expensive, slow, and resource intensive. Therefore, computational methods are highly desirable as an alternative to expedite the large-scale identification of druggable proteins (DPs); however, the existing in silico predictor’s performance is still not satisfactory.

**Methods:**

In this study, we developed a novel deep learning-based model DPI_CDF for predicting DPs based on protein sequence only. DPI_CDF utilizes evolutionary-based (i.e., histograms of oriented gradients for position-specific scoring matrix), physiochemical-based (i.e., component protein sequence representation), and compositional-based (i.e., normalized qualitative characteristic) properties of protein sequence to generate features. Then a hierarchical deep forest model fuses these three encoding schemes to build the proposed model DPI_CDF.

**Results:**

The empirical outcomes on 10-fold cross-validation demonstrate that the proposed model achieved 99.13 % accuracy and 0.982 of Matthew’s-correlation-coefficient (MCC) on the training dataset. The generalization power of the trained model is further examined on an independent dataset and achieved 95.01% of maximum accuracy and 0.900 MCC. When compared to current state-of-the-art methods, DPI_CDF improves in terms of accuracy by 4.27% and 4.31% on training and testing datasets, respectively. We believe, DPI_CDF will support the research community to identify druggable proteins and escalate the drug discovery process.

**Availability:**

The benchmark datasets and source codes are available in GitHub: http://github.com/Muhammad-Arif-NUST/DPI_CDF.

## Introduction

The Human Genome Project has enabled the discovery of new drug targets by identifying macromolecules likewise genes and proteins that are often involved in disease processes [1]. Proteins are the most common druggable targets for drug development [2] because approximately 95% of known drug targets are proteins and over 92% of known drug-target interactions involve these organic molecules [3]. The involvement of proteins in biological processes are essential for the understanding of cellular functions. Proteins can be broadly categorized into enzymes, receptors, ion channels, transporters and structural proteins based on their highly diverse structure and functions [4]. Among these protein classes druggable proteins (DPs) has unique properties that make them attractive target for the drug discovery for the treatment of many diseases, including cancer, genetic disorders, chronic disease, blood pressure, cardiovascular diseases, etc [5]. Analyzing the biochemical characteristics DP sequences, for example its binding affinity or enzymatic activity could help the researcher to provide insights about the protein interaction with other molecules inside the cell [6]. Thus, investigating about DPs and non-DPs is crucial to accelerate the drug development process for curing multiple diseases.

In recent decades, the researchers have been characterizing the DPs through wet-lab experiments such as mass spectrometry, X-ray crystallography, nuclear magnetic resonance (NMR), etc [7, 8]. These wet lab experiments for determining DPs and non-DPs are precise, but time-consuming, resource intensive and expensive due to the nature of experiments as well as the huge abundance of un-annotated proteins in databases. Moreover, development pipeline for a novel drug can be a long and expensive process, with an average development time of over 12 years and a cost of around 2.6 billion USD [9]. Furthermore, only a small percentage of drug development plans eventually result in licensed drugs, with estimates ranging from 4% to 12% [10, 11]. Hence, computational techniques led the researchers to use machine learning and deep learning algorithms as an alternative for analyzing large-scale druggable proteins data with improved accuracy.

Over the past few decades, considerable research attention has been directed toward a variety of computational methods to identify the distinctive characteristics of DPs vs. non-DPs. For example, DrugMiner [12], Sun et al. [13], GA-Bagging-SVM [5], DrugHybrid_BS [14], Yu et al. [15], XGBDrugPred [16], MS Iraji et al. [17] and SPIDER [18] are the proposed predictors for discriminating DPs from non-DPs. The pioneering work along this line was conducted by Jamali et al. [12] in 2016 and constructed a bioinformatics protocol called DrugMiner for the prediction of DPs, using multiple discrete features in conjunction with neural network classifier. The proposed model achieved over 92.10% accuracy (ACC). However, loss of sequence order information and sequence-length effects are the main shortcomings of the proposed method [19]. Afterword, Lin et al. [5] enhanced the performance by developing GA-Bagging-SVM for DPs prediction. Lin et al. first extracted the local and global feature vectors using reduced sequences, pseudo amino-acid-composition (PseAAC) and dipeptide composition (DPC). Then optimal features were selected through genetic algorithm (GA) and proposed support vector machine (SVM) based model by bagging-based ensemble strategy. Similarly, Gong et al. [14] designed hybrid-based predictor called DrugHybrid_BS using grouped amino-acid-composition, monoDIKgap and cross-variance with ensemble learning engine and achieved 97% of accuracy. Furthermore, Yu et al. [15] developed the first deep learning model to improve the overall performance of the DPs by incorporating sequence and dictionary features with ensemble convolutional recurrent neural network model(CNN-RNNs). The Yu’s model attained the 89.80% of ACC and 0.799 MCC on independent dataset. Recently, R.Sikander et al. [16], proposed machine learning model called XGBDrugPred by utilizing group di-peptide composition (DPC), reduced amino acid composition and serial-PseAAC features with extreme gradient boosting classifier. More recently, P Charoenkwan et al. proposed an effective meta-learning based classifier SPIDER, stacked predictor of druggable proteins, which predicted DPs and non-DPs with high accuracy than other existing methods [18]. Table 1 summarized the precedents of druggable protein prediction from literature.

**Table 1:** Summary of the existing works on druggable protein prediction

Method/tool	Dataset used	Feature set^a^	Proposed model^b^	Evaluation method^c^
DrugMiner [12]	Jamali et al.	AAC, PCP, DPC	NN	5CV
Sun et al. [13]	Jamali et al.	CTD	NN	5CV/IND
GA_Bagging_SVM [5]	Jamali et al.	PAAC, DPC, RC	SVM	5CV
DrugHybrid_BS [14]	Jamali et al.	monoDIKgap, CC, GAAC	SVM	5CV/IND
Yu et al. [15]	Jamali et al. and Yu et al.	DPC, TPC, Dictionary, CTD	CNN, RNN	5CV/IND
XGB DrugPred [16]	Jamali et al.	RAAAC, S-PseAAC, GDPC	XGB	10CV
SPIDER [18]	Jamali et al. and Yu et al.	AAC, CTD, RC, APAAC, PAAC, DPC	SVM	10CV/IND
DPI_CDF (our method)	Jamali et al. and Yu et al.	CPSR, NQLC, HOG-PSSM	CDF	10CV/IND

^a^AAC: amino-acid-composition, DPS: dipeptide propensity score, DPC: dipeptide composition, TPC: tripeptide composition, CTD: composition transition and distribution, CC: cross covariance, CPSR: component protein sequence representation, GAAC: grouped AAC, GDPC: grouped DPC, HOG-PSSM: histogram of oriented gradient position specific scoring matrix, NQLC: Normalized qualitative characteristics, APAAC: amphiphilic pseudo AAC, PAAC: pseudo AAC, PCP: physicochemical properties, RAAAC: reduced alphabet amino acid composition, S-PseAAC: serial pseudo amino acid composition

^b^NN: neural networks, SVM: support vector machine, CNN-RNN: convolutional-neural-network and recurrent-neural-network, XGB: extreme gradient boosting, CDF: cascade deep forest

^c^5-fold cross-validation (5CV); 10-fold cross-validation (10CV); independent test (IND)

Although each of the above mentioned predictors in (Table 1) has demonstrated a significant contribution to the prediction of DPs, however challenges remain unsolved. For instance, many existing predictors have relied upon the conventional sequence-based feature including mono-, di-, tri-peptide composition, and physicochemical properties. But these methods were unable to explore the evolutionary profile and structural properties of druggable protein sequence. Secondly, only two previous studies (Yu et al. [15], SPIDER [18]) performed an independent test evaluation to verify the generalization capability of their proposed methods. Thirdly, the overall performance of the previous models for DP prediction was not satisfactory indicating the room for improvement in the prediction capability. In the present article, we proposed a machine learning based predictor DPI_CDF for highly accurate identification of DPs and non-DPs based on novel combination of evolutionary-, physicochemical- and sequence-based feature of protein sequence. Our contribution can be briefly summarized as follows:

- We proposed new set of feature descriptor to capture the evolutionary-, sequential- and physicochemical-based patterns from a given protein sequence. Then, we hybridized this set of features to incorporate the local and global intrinsic properties of protein sequence.
- We proposed DPI_CDF, a novel model based on cascade deep forest (CDF) to predict druggable proteins with superior performance on existing training and testing benchmark datasets.
- We used interpretable t-SNE and SHAP methods to show the visual representation of the proposed features and their importance in the prediction task..

## Materials and methods

**Fig. 1:**
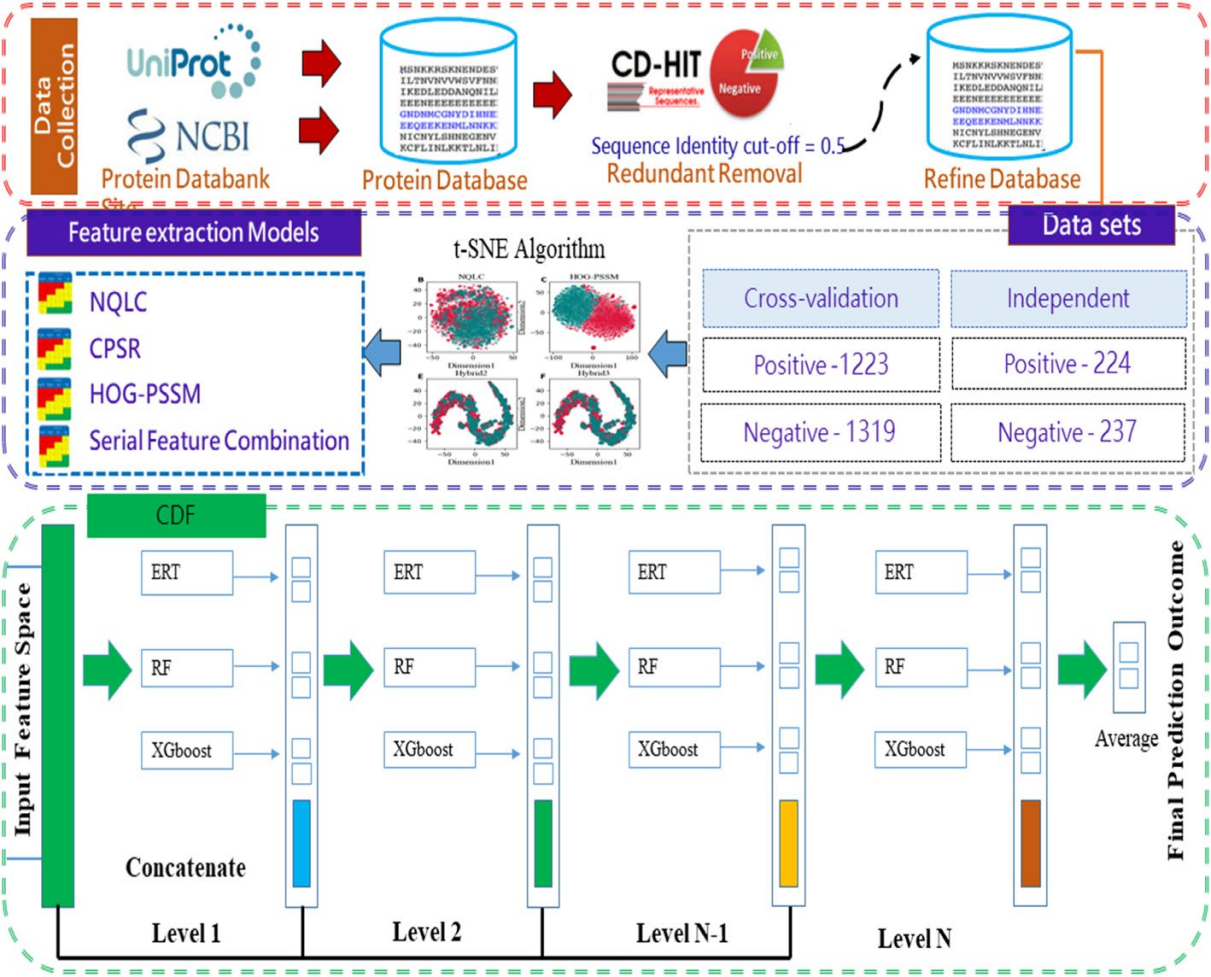
The schematic diagram of the proposed DPI_CDF

To develop DPI_CDF, we considered existing benchmark datasets of protein sequence that are already published in literature. Then we encoded the biological protein sequences into fixed length feature vector based on the compositional, physicochemical, and evolutionary properties of amino acids. Then, machine learning models were developed for the prediction of druggable protein. Finally, we evaluate the proposed model based on cross-validation and compared the performance of the proposed model against exiting methods. Figure  1 depicts the schematic diagram of the workflow for the development of DPI_CDF.

### Dataset collection

We considered the available dataset from Jamali et al. [12]. This dataset contains 1223 sequence that were considered as druggable protein sequence. It also contains 1319 sequence that are considered as non-druggable. We consider this dataset to develop machine learning model for DPI_CDF. Moreover, we consider another dataset from Yu et al. study [15] as independent dataset to determine the generalization power of the trained predictor. The independent dataset contains total of 461 sequence, of which 224 were labelled as druggable and 237 were labeled as non-druggable. Table 2 summarizes the number of samples from both datasets.

**Table 2:** Dataset summary

Dataset	Total sequence	(N_Pos, N_Neg)^a^
$DP_{train}$	2542	(1223, 1319)
$DP_{ind}$	461	(224,237)

^a^N_Pos, N_Neg represent the total number of positive and negative sequences, respectively

### Feature encoding

Feature encoding schemes are challenging task used to formulate a biological sequence into fixed length numerical feature [19–21]. In the present work, we considered physiochemical, compositional, and evolutionary-based algorithms to tackle this problem. The details of each feature descriptor are explained below.

#### Position-specific scoring matrix representation of druggable protein

The evolutionary conserved reign of amino acid residues is encoded by a technique called position-specific scoring matrix (PSSM). It has been observed that PSSM has been successfully improved the model prediction in divers bioinformatics problems for example prediction of protein folding [22], antifreeze protein identification [23] and prediction of DNA-binding protein [24]. Motivated by these precedents, we considered PSSM to encode DP sequences into feature vector. PSSM generates the corresponding feature-space of 20 attributes and M rows for an input sequence in PSI-BLAST [25] PSI-BLAST compare the DP protein evolutionary information with default parameters in Swiss-Prot databank [26]. Then, the obtained PSSM is normalized by the following mathematical formula:

1 $$\begin{aligned} f(a)=\frac{1}{1+\exp (-a)} \end{aligned}$$

where a represent the actual value of PSSM. Then we considered the PSSM from each protein to generate feature vector.

#### Histogram of oriented gradient-based extraction of PSSM

Histogram of Oriented Gradient (HOG) is widely used as a feature extractor for object detection problem in computer vision [27, 28]. The HOG-based methods provided better results compared to the existing wavelet-based methods for extracting feature from input image [29, 30]. In this study, we consider the PSSM to retain the biological evolutionary information of a sequence and applied HOG encoding method for transforming the PSSM into an HOG-PSSM. We briefly describe the steps for generating HOG based feature from protein sequence below: Firstly, we calculated the horizontal gradient $G_x(i,j)$ and vertical gradient $G_y(i,j)$ of the PSSM image by following equations:

2 $$\begin{aligned} G_x(i,j)= & {} \left\{ \begin{array}{ll} PSSM(i+1,j)-0,i=1, \\ PSSM(i+1,j)-PSSM(i-1,j),1<i<20, \\ 0-PSSM(i-1,j),i=20 \end{array} \right. \end{aligned}$$

3 $$\begin{aligned} G_y(i,j)= & {} \left\{ \begin{array}{ll} PSSM(i,j+1)-0,j=1, \\ PSSM(i,j+1)-PSSM(i,j-1),1<j<L, \\ 0-PSSM(i,j-1),j=L \end{array} \right. \end{aligned}$$

Then, we determined the magnitude as well as the direction of gradient based on the following equation:

4 $$\begin{aligned} G(i,j)= & {} \sqrt{G_x(i,j)^2+G_y(i,j)^2}, \end{aligned}$$

5 $$\begin{aligned} \Theta (i,j)= & {} \tan ^{-1}\left[ \frac{G_x(i,j)}{G_y(i,j)}\right] . \end{aligned}$$

where *G*(*i*, *j*) and $\Theta (i,j)$ are the gradient magnitude and gradient direction matrices of $L\times 20$ size. Then, we decomposed the image into fixed sized connected region called “cells”. Each cell retained the feature set of magnitude and direction of graident inside the sub-matrix.

6 $$\begin{aligned} G_{m,n}(u,v)= & {} G\left( 5\times m+1+u,n\times \frac{L}{4}+1+v\right) \end{aligned}$$

7 $$\begin{aligned} \Theta _{m,n}(u,v)= & {} \Theta \left( 5\times m+1+u,n\times \frac{L}{4}+1+v\right) \end{aligned}$$

Here *m*, *n* denotes the subscripts of sub-matrix $(0\le m\le 2,0\le n\le 2)$ and *u*, *v* denote the subscripts within sub-matrix $(0\le v\le 9,0\le v\le L/2-1)$. Based on this, each sub-matrix generates 16 different histogram channels. Finally, the resultant feature vector from HOG-PSSM for each protein sequence was of 256 (16*16).

#### Normalized qualitative characteristic feature

Qualitative characteristics feature (QLC) [31] considers the physicochemical properties of proteins which are distributed globally in a protein sequence. QLC considered hydrophobicity, charge, predicted secondary structure, polarizability, polarity, normalized Van der Waals volume, and solvent accessibility as the seven physicochemical attributes of the AA residues to categorize them into three groups [31] (Details are in Additional file 1: Table T1). The QLC descriptor encode the composition, distribution and residue wise transition of the protein based on three indexes, namely C (composition) index, D (distribution) index, and T (transition) index. Therefore it is also named as composition, transition and distribution (CTD) [32]. The C index characterizes the percent composition of each group of AA residues (based on physicochemical properties) in protein sequence; the T index (transition) signifies the transition likelihood between two adjacent residues of proteins associated with dissimilar properties; and the D index computes the distribution of AA residues along the sequence of each group in percent (25%, 50%, and 75% or 100%), respectively [33]. For each protein, C, T and D index generated 21, 21, and 105 dimensions of features, respectively. Thus, the resultant dimension of the feature was 147 for each protein sequence [32]. Then we normalized the values within the range of [0, 1] using the following formulation to generate normalized QLC (NQLC):

8 $$\begin{aligned} y_{i}=\left( \frac{x_{j}-{\bar{x}}}{std(x)}\right) \end{aligned}$$

Where $x_{i}$ denotes the physicochemical features values of $j\_{th}$ (j=1, 2, 3 â€¦20) AA residues. $\bar{x}$ denote the mean value and *std*(*x*) denote the deviation from mean of 20 AAs. $y_{i}$ represent the resultant normalized value.

#### Composite protein sequence representation

Composite Protein Sequence Representation (CPSR) descriptor is adopted to encode the prominent physicochemical properties from DPs sequences. The AA residues in proteins possesses unique physiochemical properties [34] that play a vital role in different protein function prediction problems [35, 36]. CPSR-derived method has also been used in our previous studies for encoding anticancer proteins, membrane proteins etc [37, 38]. We have used seven different types of physicochemical properties of DP sequence (Table 3 ).

**Table 3:** CPSR-based feature encoding

Feature space	Number of features
Amino acid composition	20
Sequence length	1
2-Gram exchange group frequency	36
Electron group	6
Rigidity	1
Flexibility	1
Irreplaceability	1
R-group	5

- Amino Acid Composition (AAC) For encoding protein sequence, AAC is considered the simplest formulation method. AAC counts the frequency of 20 residues in a proteins sequence and normalized its values. Resultantly, ACC generated a 20D vector of protein sequence.
- Sequence Length (L) The total number of native AAs in the given protein sequence is defined as L.
- 2-Gram Exchange Group Frequency The composition of the bi-gram exchange group plays a crucial functions in encoding the protein sequence. The exchange groups consider broad categories of AA residues that form clusters based on evolutionary effects [39]. Thus, by computing the frequency of each possible bi-gram pair, thirty six features of 36-D were generated from its equivalent 6-letter exchange group of AAs. We have provided more detail about cluster pairs of AA in Additional file 1: Table T2.
- Electron Group Based on the electron properties of AA, the 20 AA molecules can be broadly divided into six groups, i.e., acceptor or donor, electrically special and neutral AA, weak electron acceptor or donor, electron acceptor or donor electron donor or acceptor [38]. For each protein sequence, we counted the number of AA from each group and represent it as a 6-D protein feature vector.
- Rigidity We encode the rigidity of each protein sequence to describe protein structure static attributes under the impact of extrinsic factors. For each AA of a protein sequence, we summed the rigidity score and normalized by protein length, generating a 1-D feature vector.
- Flexibility “The flexibility of protein occurs universally at the level of AA side-chains and crucial for catalysis and binding function” [39]. For each AA of a protein sequence, we summed the flexibility score and then normalized by protein length, generating a 1-D feature vector.
- Irreplaceability The irreplaceability is a response to mutation deterioration during the evolution of life. To compute the irreplaceability of AA residues in protein, we summed the flexibility score and then normalized by protein length, generating a 1-D feature vector.
- R-group The AA residues in a protein sequence possess a unique chemical side chain but similar functional group. The R-group categorize the druggable protein sequence based on sub-families of AA’s and generate a 5D feature vector. The five categories are provided in the Additional file 1: Table T3.

#### Hybrid feature composition

Single set of feature may fail to capture enough attributes from protein sequence to build a generalized model [40]. In order to bring better complimentary information from several sets of feature vectors, feature hybridization is a crucial strategy [41]. Inspired by this, we adopted a serial feature hybridization technique to enhance the prediction capability of the learning algorithm. We merged HOG-PSSM, NQLC, and CPSR encoders to propose hybrid features for the model development. We considered three different hybrid features sets namely Hybrid1, Hybrid2 and Hybrid3. Hybrid1 combined the evolutionary profile and physicochemical based-feature of CPSR and HOG-PSSM and generate a feature space of 327D. Hybrid2 combined the compositional and evolutionary profile-based feature of NQLC and HOG-PSSM to form a feature space of 403D. Hybrid3 considered all features from CPSR, NQLC and HOG-PSSM to encode the protein sequence generating feature vector of 474D.

### Cascade deep forest-based predictor development

**Fig. 2:**
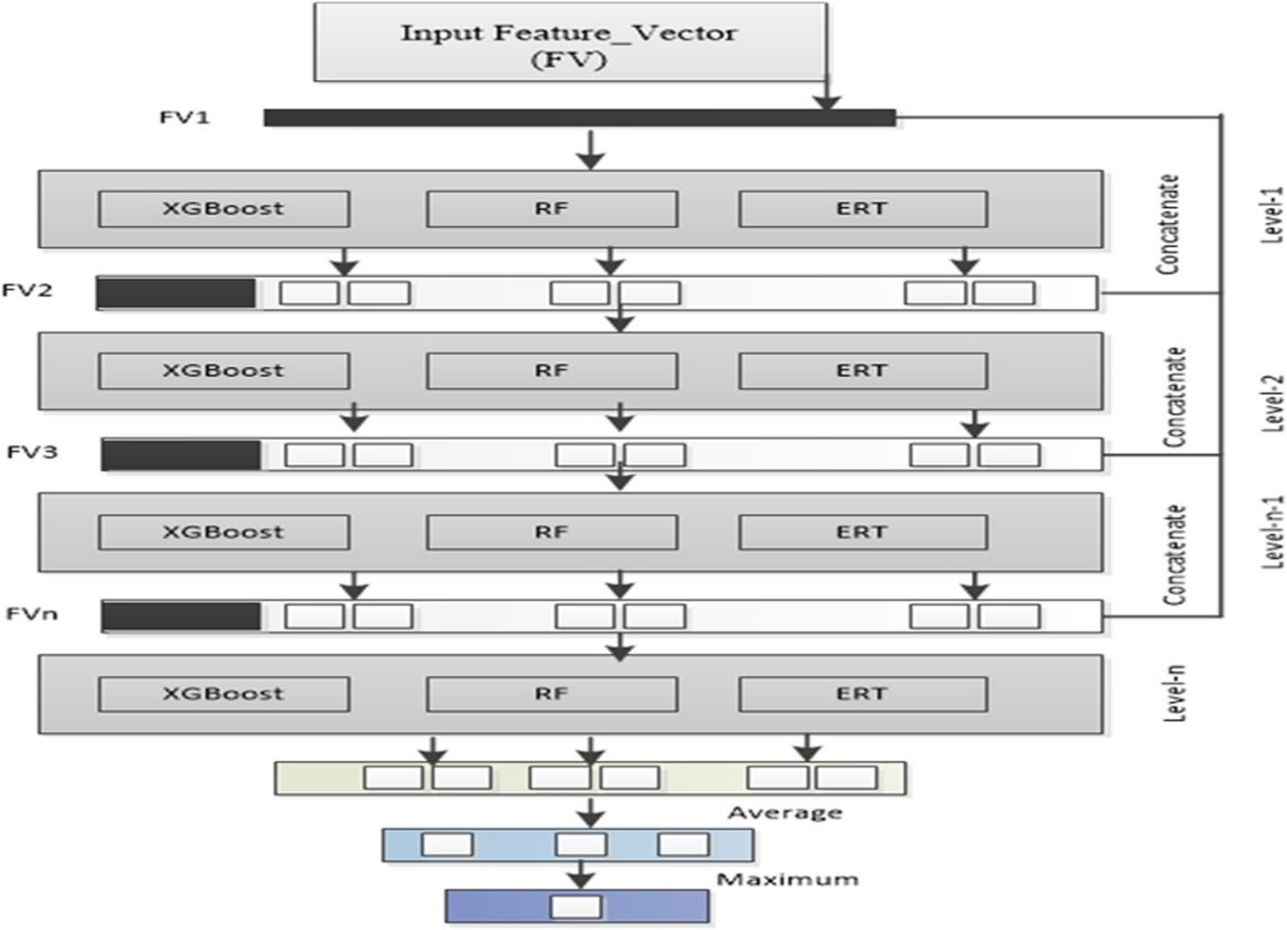
The proposed architecture of DPI_CDF classifier

The cascade deep forest (CDF) is an ensemble-based framework inspired by Zhou et al. [42] model, to the serves as a substitute for deep neural networks (DNNs) [43]. In recent research, CDF model became a has become a dominant learning algorithm in wide range of domains like pattern recognition [44, 45], and bioinformatics [46]. CDF model structure is an ensemble of trees hierarchically sequenced in multiple layers [47]. The top-down architecture of CDF enables the classifier ideal for training even limited number of samples [48]. Furthermore, Zhou and Feng pinpointed in their pioneering work that DF is much easier in tuning the hyperparameter compare to DNN [48]. Considering this, an improved version of CDF were developed containing an ensemble of RF [49], XGBoost [50], and extremely randomized trees (ERT) classifiers [51] to build DPI_CDF. Each layer of DPI_CDF is composed of four learners of XGBoost, RF and ERT machine learning classifiers that take the feature-vector of the previous layer. The previous level’s class probability is then passed on to the next layer. In order to produce the augmented attributes, the related heterogeneous feature vectors are merged, averaged and the maximum probability values is generated as output. The hyper parameter of the models were tuned using GridSearchCV and the selected parameters are added in Additional file 1: Table T6. The node split attributes were selected by randomly selecting features, where $\sqrt{d}$ is the total number of features. Figure 2 shows the layer-by-layer architecture of the DPI_CDF.

### Performance evaluation metrics

To measure the prediction performance of binary class, we use four performance evaluation metrics: sensitivity (SEN), accuracy (ACC), specificity (SPE), and Matthew’s correlation coefficient (MCC). These measures are mathematically formulated as follows:

9 $$\begin{aligned} ACC= & {} \frac{(tp+tn)}{(tp+tn+fp+fn)} \end{aligned}$$

10 $$\begin{aligned} SEN= & {} \frac{tp}{tp+fn} \end{aligned}$$

11 $$\begin{aligned} SPE= & {} \frac{tn}{tn+fp} \end{aligned}$$

12 $$\begin{aligned} MCC= & {} \frac{tp\cdot tn-fp\cdot fn}{\sqrt{(tp+fp)(tp+fn)(tn+fp)(tn+fn)}} \end{aligned}$$

In the above Eqs. (09–12), tp represents the correctly predicted DPs,tn represents the correctly predicted non-DPs. Whereas fn represent the incorrectly predicted DPs and in contrast the fp represent the non-DPs predicted incorrectly by the model. The above-mentioned performance metrics are threshold dependent. In order to shed light the proposed model efficacy an independent evaluation measure receiver operating characteristic (ROC) curve (AUC) was adopted [52].

### Model evaluation

The performance of machine learning models were evaluated based on k-fold cross-validation (CV). In k-fold CV, benchmark dataset was divided into k subsets (folds) of nearly same size. Of the all the k folds, the k-1 folds were used for training the model and the remaining one is taken for testing the model [53]. In this work, we used 10 fold CV to evaluate the generalization power of the model based on Jamal et al. dataset [12] and an independent dataset (Yu’s dataset [15] ) was used for examining the model performance.

## Results and discussion

### Performance of DPI_CDF using various feature descriptors on training $(DP_{train})$ and testing $(DP_{ind})$ dataset

In this section, we analyzed the efficacy of four classification algorithms including multi-layer perceptron (MLP), ERT( Extra Tree Classifier), XGBoost and DPI_CDF using various single-view descriptors, i.e., CPSR, NQLC, and HOG-PSSM and series combination of multi-view descriptors i.e., Hybrid1, Hybrid2 and Hybrid3. The classifiers were trained using 10-fold CV on $DP_{train}$ dataset and evaluated on $DP_{ind}$ independent dataset with five evaluation measures AUC, SEN, SPE, MCC and ACC. We can comprehensively analyze several observations from Table 4 as follows; First, in case of individual feature space, HOG-PSSM produces outstanding prediction results on cascade deep forest classifier which are mean ACC of 94.77% and MCC of 0.895. The second-best performer on HOG-PSSM is XGBoost learning engine which attain 93.63% of ACC and 0.876 of MCC respectively. However, in contrast it achieves worst predictions on MLP classifier i.e., ACC=79.23% and MCC =0.594. The CPSR encoding method comparatively generates satisfactory results on classifiers. Secondly, to improve the prediction performance of the proposed model, feature fusion strategy was employed. It is clear from empirical results in Table 4 that our proposed DPI_CDF model train on hybrid features particularly Hybrid3 (HOG-PSSM+CPSR+NQLC) features produce superior results than single-view descriptors on all evaluation indicators ACC, MCC, SEN and SPE. The highest success rates in terms of ACC=99.23% and MCC=0.99 are obtained by DPI_CDF using Hybrid5 feature set. On the other hand, ERT classifier performed over all poor predictions on the hybrid feature sets. We also performed the 5-,-6,and -8fold cross validation on the training dataset and the results are highlighted in Additional file 1: Table T4. Using 10-fold CV we got the best results.

**Table 4:** Performance of various feature descriptors on DPtrain benchmark dataset using 10-fold CV test

Classifier	Feature vector	ACC (%)	SEN (%)	SPE (%)	MCC	AUC
MLP	CPSR	88.08	86.63	86.63	0.770	0.932
	NQLC	87.14	87.27	87.04	0.750	**0.958**
	HOG-PSSM	79.23	85.69	73.24	0.594	0.872
	Hybrid1	88.20	87.85	88.56	0.776	0.950
	Hybrid2	87.69	85.31	89.92	0.760	0.954
	Hybrid3	**89.98**	**86.63**	**93.10**	**0.810**	0.953
ERT	CPSR	**87.34**	**85.47**	**89.09**	**0.751**	**0.934**
	NQLC	86.08	83.91	88.1	0.724	0.928
	HOG-PSSM	82.06	83.89	80.37	0.643	0.878
	Hybrid1	80.17	82.00	78.48	0.605	0.883
	Hybrid2	83.87	83.41	84.31	0.678	0.920
	Hybrid3	83.16	82.99	83.33	0.664	0.916
XGBoost	CPSR	87.77	85.63	89.76	0.759	0.944
	NQLC	88.71	85.88	91.35	0.778	0.949
	HOG-PSSM	**93.63**	**94.10**	93.18	**0.876**	**0.986**
	Hybrid1	93.62	93.20	**94.01**	0.873	0.968
	Hybrid2	92.76	93.20	92.34	0.859	0.969
	Hybrid3	93.59	93.21	93.93	0.873	0.968
DPI_CDF	CPSR	90.09	87.52	92.49	0.806	0.956
	NQLC	89.22	85.96	92.26	0.788	0.949
	HOG-PSSM	94.77	93.64	95.82	0.895	0.969
	Hybrid1	99.13	98.52	**99.69**	0.982	**0.999**
	Hybrid2	99.21	98.93	99.46	0.984	0.998
	Hybrid3	**99.33**	**99.02**	**99.62**	**0.986**	0.998

In order to determine the proposed model prediction power, an independent or blind test is generally conducted. Table 5 illustrates the performance of all classifiers using various feature encoding methods on independent dataset. We can easily see that DPI_CDF with Hybrid1 (HOG-PSSM + CPSR) feature set achieve best performance on all evaluation metrics i.e., ACC=95.01%, MCC=0.900, SPE=93.24, AUC=0.986, and SEN=96.87%. The second best model is XGBoost classifier that achieved comparatively consistent results than MLP and ERT on various feature encoding schemes. Moreover, we have added the confusion matrix(TP, TN, FP, and FN) predictions of the proposed DPI-CDF model in Additional file 1: Table T4 and T5.

**Table 5:** Performance of various feature descriptors on independent dataset DPind

Classifier	Feature vector	ACC (%)	SEN (%)	SPE (%)	MCC	AUC
MLP	CPSR	89.37	83.03	**95.35**	**0.792**	**0.940**
	NQLC	**90.23**	86.60	93.67	0.805	0.937
	HOG-PSSM	78.09	73.66	82.27	0.562	0.845
	Hybrid1	86.98	**91.51**	82.70	0.743	**0.940**
	Hybrid2	88.93	86.16	91.56	0.779	0.936
	Hybrid3	89.37	88.39	90.29	0.787	0.946
ERT	CPSR	**86.55**	**79.46**	**93.24**	**0.736**	**0.892**
	NQLC	83.08	75.00	90.71	0.667	0.880
	HOG-PSSM	77.87	73.66	81.85	0.557	0.838
	Hybrid1	72.45	70.08	74.68	0.448	0.799
	Hybrid2	77.86	74.55	81.01	0.557	0.865
	Hybrid3	75.92	68.75	82.70	0.520	0.855
XGBoost	CPSR	87.85	83.03	92.40	0.759	0.912
	NQLC	87.85	82.14	93.24	0.760	0.917
	HOG-PSSM	89.37	**88.83**	**89.87**	**0.787**	**0.964**
	Hybrid1	89.15	**88.83**	89.45	0.782	0.936
	Hybrid2	89.37	**88.83**	**89.87**	**0.787**	0.941
	Hybrid3	89.15	**88.83**	89.45	0.782	0.936
DPI_CDF	CPSR	87.41	87.94	86.91	0.748	0.927
	NQLC	85.24	79.91	90.29	0.707	0.893
	HOG-PSSM	94.14	96.42	91.98	0.883	0.980
	Hybrid1	**95.01**	**96.87**	**93.24**	**0.900**	**0.986**
	Hybrid2	94.36	96.86	91.98	0.888	0.977
	Hybrid3	94.57	96.87	92.40	0.892	0.978

**Fig. 3:**
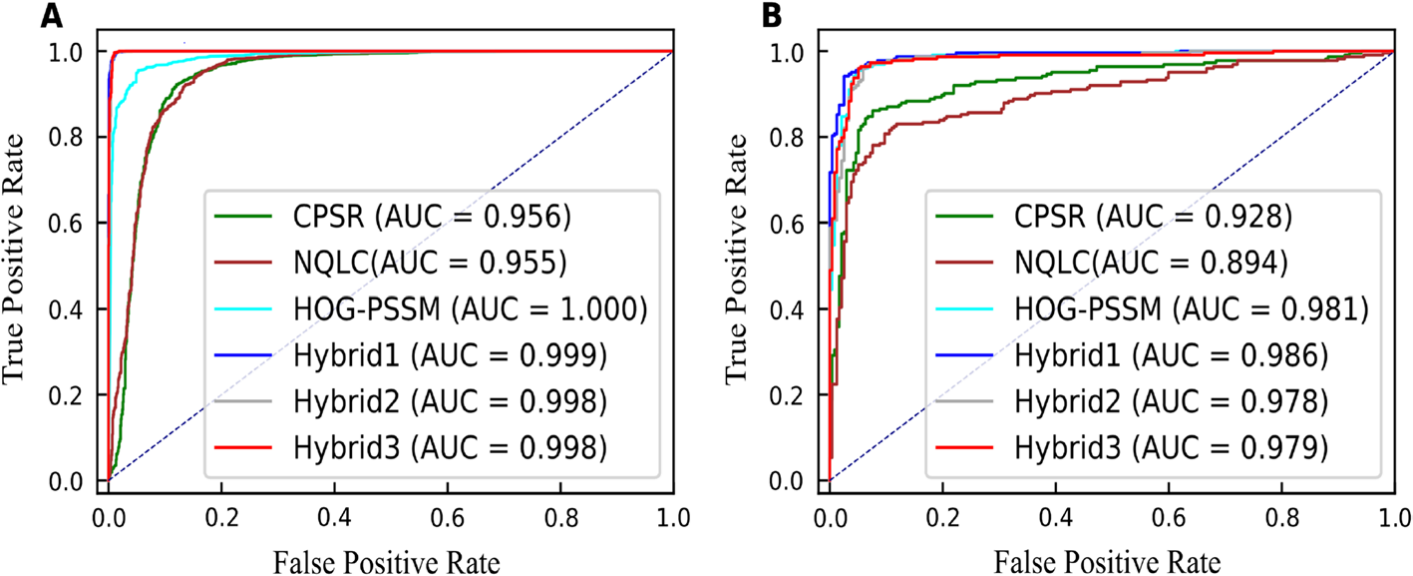
ROC curves of DPI_CDF model using various feature encoding methods on the training (**A**) and testing (**B**) datasets

We also generated the receiver operating characteristics (ROC) curve for the proposed DPI_CDF model on training and independent set (Fig. 3). We can observe that the model with Hybrid3 based feature combination achieved the highest AUC for training and test set with 0.998 and 0.979, respectively.

### Comparison with previous predictors

We comprehensively compared DPI_CDF with previously developed sequence-based computational models including DrugMiner [12], Sun’s Method [13], GA-Bagging-SVM [5], YU’s Method [15], XGB-DrugPred [16], and SPIDER [18] for characterizing and identifying DPs and non-DPs. It is worth noting that among these approaches, only two predictors i.e. Yu’s Method [15] and SPIDER [18] were examined on both training $DP_{train}$ and testing $DP_{ind}$ datasets. The comparison outcomes of past studies over training and testing datasets are reported in Tables 6 and 7, respectively. The high prediction value of each criterion is presented in bold fonts. It is clear from Table 6 that DPI_CDF achieved highest performance on training dataset in terms of ACC 99.13%, MCC of 0.982, SPE of 99.69%, F-score of 0.999 and SEN of 98.52% which are 7.23%, 14.3%, 5.49%, 8.5% and 9.02% higher than recent state-of-the-art SPIDER method. Furthermore, to demonstrate the generalization power of DPI_CDF on unseen data, independent test set results are reported in Table 6. The prediction outcomes in Table 6 reveals that DPI_CDF attained ACC of 95.01%, MCC of 0.900, SPE of 93.24%, F-score of 0.949 and SEN of 96.87%. Our proposed protocol showed superior performance in term of evaluation indexes ACC of 4.31%, MCC of 8.4%, F-score of 5% and SEN of 11.17%, except little decrease in term of SPE than SPIDER. From aforementioned discussion, it can be concluded that the proposed method for determining the proteins druggability is far superior to all the available computational methods.

**Table 6:** Performance comparison of DPI-CDF predictor with existing methods on training dataset $DP_{train}$

Predictor	Algorithm	ACC (%)	SEN (%)	SPE (%)	MCC	F-score
DrugMiner	NN	92.1	92.8	91.34	0.841	0.924
Sun’s Method	NN	91.93	N/A	N/A	N/A	N/A
GA-Bagging-SVM	SVM	93.78	92.86	94.45	0.878	0.935
Yu’s Method	CNN-RNN	90	89	N/A	0.8	0.896
SPIDER	SVM	91.9	89.5	94.2	0.839	0.914
XGB-DrugPred	XGBoost	94.86	93.75	95.74	0.89	0.963
DPI_CDF (our method)	CDF	**99.13**	**98.52**	**99.69**	**0.982**	**0.999**

N/A: Not available in the literature

**Table 7:** Performance comparison of DPI-CDF predictor with existing state-of-the-art methods on independent dataset $DP_{ind}$

Predictor	Algorithm	ACC (%)	SEN (%)	SPE (%)	MCC	F-score
Yu’s method	CNN-RNN	89.8	84.8	89.5	0.799	0.889
SPIDER	SVM	90.7	85.7	95.4	0.816	0.899
DPI_CDF (our method)	CDF	**95.01**	**96.87**	**93.24**	**0.90**	**0.949**

**Fig. 4:**
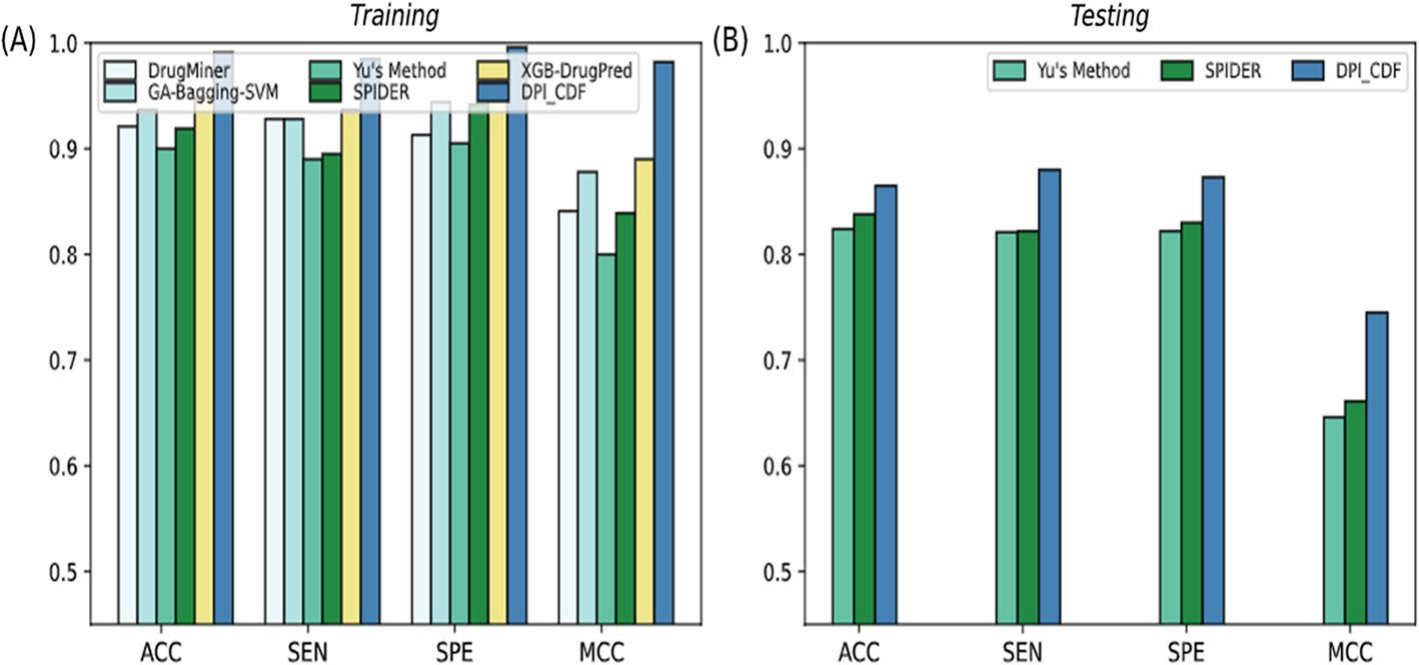
Performance comparison of DPI_CDF with existing DPs predictors over training (**A**) and testing (**B**) dataset

Figure 4 highlights the performance evaluation metrics for DPI_CDF along with other existing methods for predicting druggable proteins. We can observe that both in training and independent set, DPI_CDF outperformed the existing methods. The proposed model attained the highest ACC and MCC of 95.01% and 0.949 respectively on independent test.

### Visual analysis and explanation of the proposed features

**Fig. 5:**
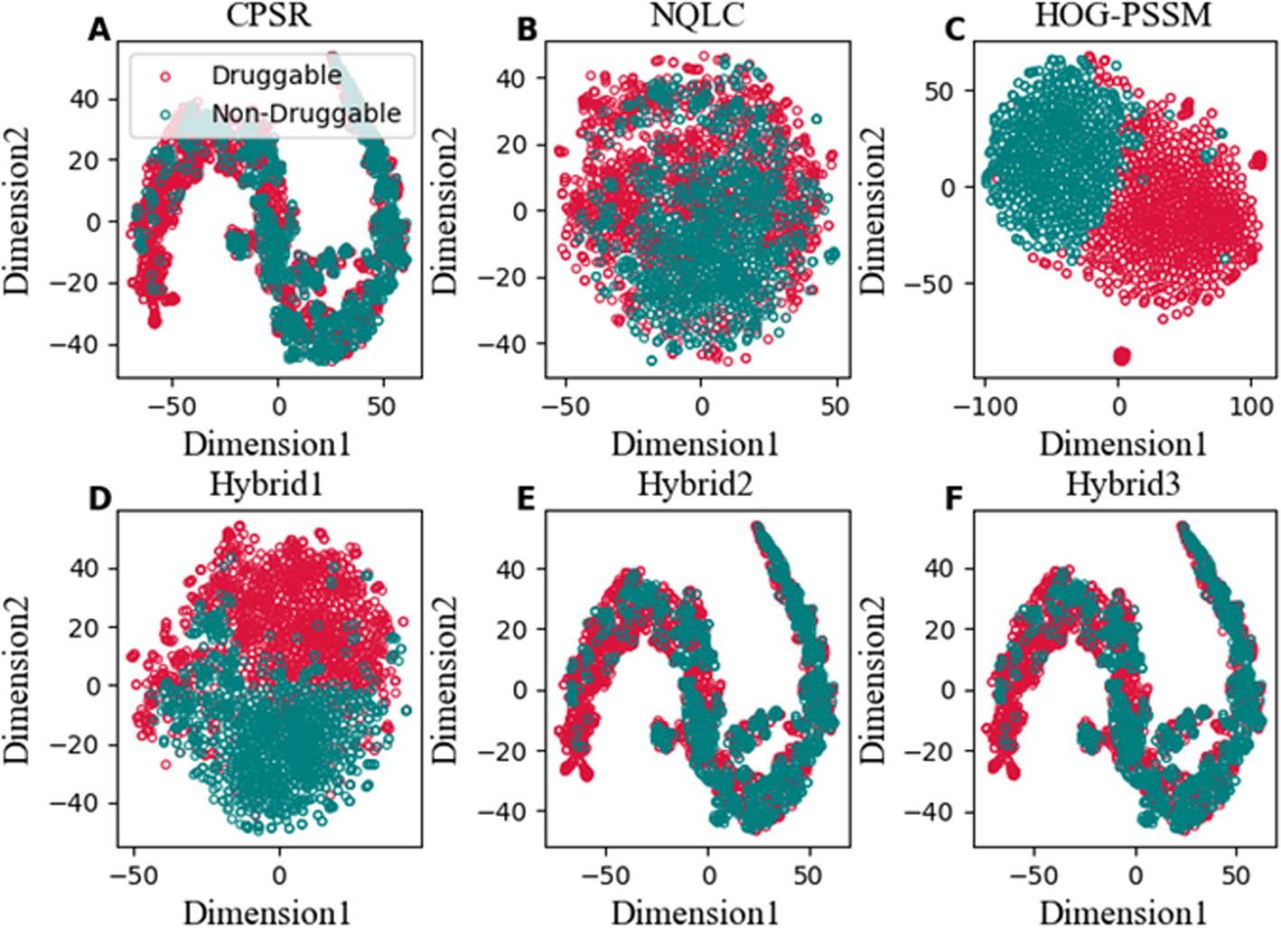
t-SNE visualization of Druggable (red) and non-Druggable (green) samples on the training dataset in a two-dimensional feature space: CPSR (**A**), NQLC (**B**), HOG-PSSM (**C**), Hybrid1 (**D**), Hybrid2 (**E**), and Hybrid3 (**F**)

In order to interpret the impact of engineered features, we used two dimension scatters plot t-SNE [54] and SHAP to visualize the distribution of extracted single-view features (CPSR, NQLC and HOG-PSSM) and multi-view features (Hybrid1, Hybrid2, and Hybrid3) on training dataset (Fig. 5).

In Fig. 5, the green dots represent the non-DPs and red dots represent DPs. Figure 5A-C are single-view descriptors, indicating that HOG-PSSM shows sharp distinction between the distribution of green and red dots (Fig. 5C) which significantly contribute to predicting DPs. Similarly, Fig. 5D–F are mixing different feature combination of (evolutionary +physicochemical) Hybrid1, (evolutionary + compositional) Hybrid2, and (evolutionary +physicochemical + compositional) Hybrid3. The plotting distribution of Hybrid1 feature in Fig. 5D seems more distinguishable than the other fused features indicate that Hybrid1 explore the biological region of DPs. The combination of physicochemical and evolutionary-based attributes is more effective in designing DPI_CDF model for DPs and non-DPs classification.

**Fig. 6:**
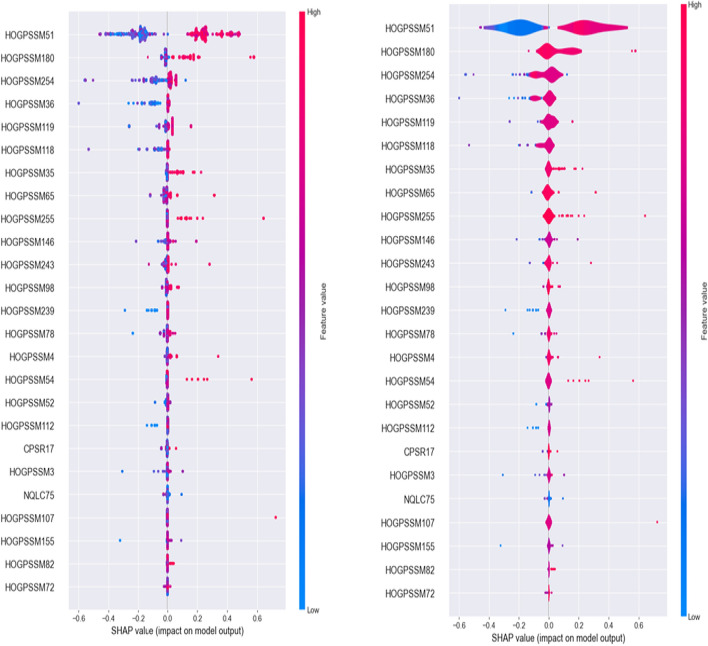
SHAP analysis for the top ranked 25 features for DPI_CDF

Furthermore, SHAP (Shapley Additive exPlanations) method [55] was used to elucidate the relative contribution of each feature in model performance (Fig. 6). It is clear from the Fig. 6 that the positive and negative SHAP values for the top ranked features favored the prediction performance of DPs and non-DPs, respectively. Majority of the top ranked features particularly HOG-PSSM51 and HOG-PSSM180 captured the key DPs attributes and had positive SAHP values, the model predicted a protein sequence as DP; otherwise a protein sequence was predicted as non-DP for negative SHAP values. We also noticed that among the toped ranked attributes, CPSR17 and NQLC75 from CPSR and NQLC, respectively contributed to boosting the performance of DPI_CDF. Thus, it is evident that feature fusion strategy helped to enhance the prediction capability of the proposed DPI_CDF model.

## Conclusion

Identification of drug targets is crucial for pharmaceutical industries to design new efficacious drugs. In this work, we have designed a novel high-throughput model, DPI_CDF, for screening proteins with druggable activity. To our best knowledge, DPI_CDF is the first ensemble-based method based on evolutionary, physiochemical and compositional feature vectors for characterizing and discriminating DPs and non-DPs. Experiment outcomes on the benchmark datasets anticipate that our proposed predictor attained superior performance in druggable target prediction and surpassed all the existing sequence-based DP prediction tools. Additionally, the DPI_CDF protocol shows excellent efficacy due to multiple reasons. (a) The new encoding schemes were designed to dig out the prominent information from the biological protein sequences. (b) Feature fusion strategy enhanced the overall performance of the model. (c) Designing a robust and efficient CDF learning algorithm for druggable protein identification.

Our work has some limitations that need to be mentioned. We considered hand curated features to encode protein sequences which requires domain expertise. We did not provide our methods as a web server or binary tool for the users. In future we will consider different combinations of novel features to encode proteins which may further improve the performance of DPI_CDF. In future we will try to collect more data and manually curate them to provide a high-quality larger dataset for this particular problem. We will also explore deep learning-based models, e.g., RNN, LSTM, B-LSTM, etc. to improve the performance of the predictor on large-scale un-annotated proteins.

### Additional file

**Additional file 1:** **Table T1.** Normalized Qualitative Characteristics (NQLC) for amino acid residues. **Table T2.** Composite Protein Sequence Representation based on property group of amino acids, (a) Exchange Group, (b) Electron Group, and (c) R group. **Table T3.** Physicochemical index values of amino acid residues. **Table T4.**DPI-CDF Model for 5-, 6-, 8-fold CV results using all features and confusion matrix. **Table T5.** DPI-CDF Model for 10-fold CV results using all features and Confusion Matrix. **Table T6.** Information of hyper parameter settings for DPI-CDF used in this study.

## Data Availability

Source code and data is shared on GitHub at: http://github.com/Muhammad-Arif-NUST/DPI_CDF
