# Supplementary material for: DPI_CDF: druggable protein identifier using cascade deep forest
Source: BMC Bioinformatics. 2024 Apr 5;25:145. doi: 10.1186/s12859-024-05744-3 (PMC11334562; doi:10.1186/s12859-024-05744-3)
Supplement: Supplementary file 1 — Additional file 1: Table T1. Normalized Qualitative Characteristics (NQLC) for amino acid residues. Table T2. Composite Protein Sequence Representation based on property group of amino acids, (a) Exchange Group, (b) Electron Group, and (c) R group. Table T3. Physicochemical index values of amino acid residues. Table T4.DPI-CDF Model for 5-, 6-, 8-fold CV results using all features and confusion matrix. Table T5. DPI-CDF Model for 10-fold CV results using all features and Confusion Matrix. Table T6. Information of hyper parameter settings for DPI-CDF used in this study. [file 12859_2024_5744_MOESM1_ESM.pdf]

Supplementary Files

Supplementary Table T1: Normalized Qualitative Characteristics (NQLC) for amino acid residues.

Amino Acids	Group1	Group2	Group3
Hydrophobicity	Polar	Neutral	Hydrophobicity
	**R,K,E,D,Q,N**	**G,A,S,T,P,H,Y**	**C,L,V,I,M,F,W**
Normalized van der Waals volume	**0-2.78**	**2.95-4.0**	**4.03-8.08**
	**G,A,S,T,P,D**	**N,V,E,C,Q,I,L**	**M,H,K,F,R,Y,W**
Polarity	**4.9-6.2**	**8.0-9.2**	**10.4-13.0**
	**L,I,F,W,C,M,V,Y**	**P,A,T,G,S**	**H,Q,R,K,N,E,D**
Polarizability	**0-1.08**	**0.128-0.186**	**0.219-0.409**
	**G,A,S,D,T**	**C,P,N,V,E,Q,I,L**	**K,M,H,F,R,Y,W**
Charge	**Positive**	**Neutral**	**Negative**
	**K,R**	**A,N,C,Q,G,H,I,L,M,F,P,S,T,W,Y,V**	**D,E**
Secondary structure	**Helix**	**Strand**	**Coil**
	**E,A,L,M,Q,K,R,H**	**V,I,T,C,W,F,T**	**G,N,P,S,D**
Solvent-accessible	**Buried**	**Exposed**	**Intermediate**
	**A,L,F,C,G,I,V,W**	**R,K,Q,E,N,D**	**M,S,P,T,H,Y**

Supplementary Table T2: Composite Protein Sequence Representation based on property group of amino acids, (a) Exchange Group, (b) Electron Group, and (c) R group.

Group	Sub-group	Amino Acid
Exchange group	e_1	KHR
	e_2	DENQ
	e_3	C
	e_4	AGPST
	e_5	ILMV
	e_6	FYW
Electron group	Electron donor	DEPA
	Weak Electron donor	VLI
	Electron Acceptor	KNR
	Weak electron Acceptor	FYMTQ
	Neutral	GHWS
	Special AA	C
R-group	Non-polar aliphatic	ALIV
	Glycine	G
	Non-polar	FMPW
	Polar uncharged	CNQSTV
	Charged	DEHKR

Supplementary Table T3. Physicochemical index values of amino acid residues

Amino Acid	Rigidity	Flexibility	Irreplaceability
A	-1.33	-3.10	0.52
C	-1.51	0.95	1.12
D	-0.20	0.42	0.77
E	-0.36	2.00	0.76
F	2.87	-0.46	0.86
G	-1.09	-2.74	0.56
H	2.26	-0.22	0.94
I	-1.74	0.42	0.65
K	-1.82	3.95	0.81
L	-1.74	0.42	0.58
M	-1.74	2.48	1.25
N	-0.20	0.42	0.79
P	1.97	-2.40	0.61
Q	-0.36	2.00	0.86
R	1.16	3.06	0.60
S	-1.51	0.95	0.64
T	-1.64	-1.33	0.56
V	-1.64	-1.33	0.54
W	5.91	-1.00	1.82
Y	2.71	-0.67	0.98

Table T4 DPI-CDF Model for 5-, 6-, 8-fold CV results using all features and confusion matrix

**Model**	**No of fold**	**TP**	**TN**	**FP**	**FN**	**ACC**	**SEN**	**SPE**	**MCC**
DPI_CDF	5	1203	1310	20	9	98.85	98.36	99.31	0.977
DPI_CDF	6	1199	1315	24	4	98.89	98.03	99.69	0.978
DPI_CDF	8	1204	1313	19	6	99.01	98.44	99.54	0.980

Note: TP: True positive; TN: True negative: FP; False positive; FN: False negative; ACC: Accuracy; MCC: Matthews correlation coefficient; SEN: Sensitivity; SPE: Specificity

Table T5 DPI-CDF Model for 10-fold CV results using all features and Confusion Matrix

Model	No of Fold	TP	TN	FP	FN	ACC	SEN	SPE	MCC
DPI-CDF	10	1205	1315	18	4	**99.13**	**99.02**	**99.62**	**0.986**

Note: TP: True positive; TN: True negative: FP; False positive; FN: False negative; ACC: Accuracy; MCC: Matthews correlation coefficient; SEN: Sensitivity; SPE: Specificity

Table T6. Information of hyper parameter settings for DPI-CDF used in this study.

**Parameter**		**Search Space**	**Best Fit**
**CDF**			
n_estimators		[50, 10, 20, 300,400]	500
max_depth		[2, 3, 4, 5]	4
n_jobs		[1]	1
learning_rate		[0.005, 0.01, 0.02, 0.03,]	0.01
**MLP**			
hidden_layer_sizes		[50 100,150, 200]	100
Activation		[relu' (Rectified Linear Unit), 'logistic' (sigmoid function), and 'tanh' (hyperbolic tangent)]	ReLu
solver		'adam', 'lbfgs', and 'sgd'	Adam
batch_size		8,16,32	16
learning_rate		[0.1, 0.01, 0.001, 0.05,]	0.01
**RF**			
n_estimators		[50, 10, 20, 300,400]	100
criterion		[gini, entropy]	gini
max_features		[log2, auto, sqrt, integer, float]	auto
min_samples_leaf		[1,2,3,4,5]	1
**XGBoost**			
n_estimators		[50, 100, 150, 200, 250]	100
learning_rate		[0.05, 0.1, 0.2, 0.3]	0.1
